# Understanding Cerebral Blood Flow Dynamics for Alzheimer's Disease Prevention Through Acute Exercise (flADex): Protocol for a Randomized Crossover Trial

**DOI:** 10.1002/brb3.70636

**Published:** 2025-10-31

**Authors:** Isabel Martín‐Fuentes, Beatriz Fernandez‐Gamez, Sol Vidal‐Almela, Alfredo Caro‐Rus, Patricio Solis‐Urra, Lucía Sánchez‐Aranda, Javier Fernández‐Ortega, Javier Sanchez‐Martinez, Andrea Coca‐Pulido, Marcos Olvera‐Rojas, Emilio J. Barranco‐Moreno, Jose D. Marin‐Alvarez, Esmée A. Bakker, Angel Toval, Darío Bellón, Alessandro Sclafani, Thomas K. Karikari, Kirk I. Erickson, Manuel Gómez‐Río, Francisco B. Ortega, Irene Esteban‐Cornejo

**Affiliations:** ^1^ Department of Physical and Sports Education, Sport and Health University Research Institute (iMUDS), Faculty of Sport Sciences University of Granada Granada, Andalucía Spain; ^2^ Faculty of Education and Social Sciences University Andres Bello Viña del Mar Chile; ^3^ AdventHealth Research Institute Neuroscience Orlando Florida USA; ^4^ Department of Primary and Community Care Radboud University Medical Center Nijmegen Netherlands; ^5^ Department of Psychiatry and Neurochemistry, Institute of Neuroscience and Physiology, The Sahlgrenska Academy University of Gothenburg Gothenburg Sweden; ^6^ Department of Psychiatry University of Pittsburgh Pittsburgh Pennsylvania USA; ^7^ Servicio de Medicina Nuclear Hospital Universitario Virgen de las Nieves Granada, Andalucía Spain; ^8^ ibs.GRANADA Instituto de Investigación Biosanitaria Granada, Andalucía Spain; ^9^ CIBER de Fisiopatología de la Obesidad y Nutrición (CIBEROBN) Instituto de Salud Carlos III Madrid Spain; ^10^ Faculty of Sport and Health Sciences University of Jyväskylä Jyväskylä Finland

**Keywords:** aging, blood biomarkers, cerebral blood flow, cerebral blood flow exercis, cognition, exercise, magnetic resonance imaging

## Abstract

**Introduction:**

Alzheimer's disease (AD) is a leading cause of disability worldwide. Alterations in cerebral blood flow (CBF) and AD blood biomarkers are fundamental at early stages of AD. Exercise shows promise in delaying physiological changes, but its mechanisms for enhancing brain health remain unclear. flADex aims to examine the acute effects of different exercise types on CBF and blood biomarkers in older adults. This protocol describes the methodology and rationale of flADex.

**Methods:**

flADex is a counterbalanced crossover trial in 20 older adults, aged 68–83 years old, with negative brain amyloid status (< 12 centiloid) who are APOEε4 noncarriers. Participants will complete a 30‐min session of each condition in a randomized order: (i) moderate‐intensity aerobic exercise (60%–70% age‐predicted maximal heart rate), (ii) moderate‐intensity resistance exercise (rating of perceived exertion: 4–6 points out of 10), and (iii) resting condition. Changes in CBF are the primary outcome and will be assessed by magnetic resonance imaging using pseudo‐continuous arterial spin labeling at pre‐ and at 3 timepoints post‐condition (starting at 20, 27, 34 min). Secondary outcomes are biomarkers of AD pathology and neurodegeneration (Aβ42, Aβ40, p‐tau217, p‐tau181, BD‐tau, GFAP, NfL) and growth factors (BDNF, IGF‐1), measured through blood samples collected at pre‐ and post‐condition (at 3, 50, 70 min). Moreover, cognitive outcomes and mood status will be measured pre‐ and post‐condition.

**Conclusion:**

flADex will highlight the acute effects of different exercise types on CBF and biomarkers before beta‐amyloid accumulation. Acute effects on CBF dynamics and blood biomarkers are expected to be greater with aerobic than resistance exercise when compared to resting. CBF is expected to vary by brain region, and biomarkers to fluctuate dynamically postexercise. This will provide critical insights into exercise's impact on vascular and molecular pathways associated with AD pathology and potential recommendations for standardized blood sampling to enhance diagnostic accuracy.

## Graphical abstract

This randomized crossover trial investigates how acute aerobic and resistance exercise influence cerebral blood flow, Alzheimer's‐related blood biomarkers, cognition, and mood in older adults. Using multimodal assessments, the flADex study aims to uncover neurovascular mechanisms for Alzheimer's prevention.

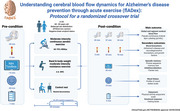

## 1 Background

Alzheimer's disease (AD) is a leading cause of disability and dependency worldwide (Prince et al. 2016), characterized by a gradual deterioration in cognitive and functional abilities. Pathophysiological signs of AD begin approximately 10–20 years before the onset of symptoms of cognitive decline (Zlokovic 2011; Long and Holtzman 2019). The accumulation of amyloid‐beta (Aβ) plaques and neurofibrillary tangles composed of hyperphosphorylated tau protein (p‐tau) are well‐established pathological hallmarks of AD. Additionally, cerebral blood flow (CBF) has gained increasing attention as a key vascular pathway in AD research. Indeed, CBF dysregulation can be observed in older adults at risk for AD, even before the appearance of Aβ accumulation or brain atrophy (Zlokovic 2011; Iadecola 2013). Disrupted CBF exacerbates neurodegeneration by upregulating the BACE1 enzyme—which produces Aβ—and by promoting the accumulation of p‐tau (Zlokovic 2011; Korte et al. 2020). This reduction in CBF may significantly contribute to cognitive decline by either triggering the amyloid cascade or amplifying Aβ production through a feedback loop (Korte et al. 2020).

The chronic neuroprotective benefits of exercise likely result from the repeated acute effects that occur during or following each exercise bout, as bodily systems respond to meet the increased metabolic demands (Swain et al. 2003; Maass et al. 2015). Observational and animal studies suggest that chronic exercise is associated with increased CBF and angiogenesis in the brain (Swain et al. 2003; Rogers et al. 1990). During aerobic exercise, CBF often follows an inverted U response, increasing at the start of exercise, plateauing, and then declining towards the end of exercise (Mulser and Moreau 2023; Mast et al. 2022). However, after exercise, the cerebrovascular effects vary across brain regions and over time, depending on the type of exercise (e.g., aerobic vs. resistance) (Mast et al. 2022; Macintosh et al. 2014; Steventon et al. 2020). Further, emerging evidence suggests that exercise may acutely alter the concentrations of neurodegenerative biomarkers (e.g., Aβ, neurofilament‐light chain [NfL]) and growth factors (e.g., brain‐derived neurotrophic factor [BDNF], insulin‐like growth factor 1 [IGF‐1]) in the bloodstream (Rodriguez‐Ayllon et al. 2024; Joisten et al. 2021). These changes are hypothesized to influence cognitive function, potentially mediating the brain health benefits of exercise (Maass et al. 2015; Rogers et al. 1990). However, the acute implications of exercise‐induced CBF changes on blood biomarkers and cognitive function following different types of exercise remain underexplored. Investigating this is also crucial for establishing recommendations on whether blood samples for biomarkers of AD pathology and neurodegeneration should be collected under standardized rest conditions prior to measurements.

Research on acute responses to exercise, using neuroimaging combined with measures of CBF, blood biomarkers, and cognitive function, is essential to reveal how exercise enhances brain health and aids in preventing or managing AD (Rodriguez‐Ayllon et al. 2024). Therefore, flADex will examine the acute effects of aerobic and resistance exercise compared to a resting condition on CBF, blood biomarkers, and their cognitive implications in older adults. This protocol describes the rationale and methodology of the flADex trial.

### 1.1 Trial Objectives

The *primary objective* is to examine the acute effects of a bout of aerobic exercise, resistance exercise, and a resting condition on global and regional CBF using cutting‐edge magnetic resonance imaging (MRI) in older adults.

The *secondary objectives* are to examine: (i) the acute effects of a bout of aerobic exercise, resistance exercise, and a resting condition on blood biomarkers of AD pathology and neurodegeneration (Aβ42, Aβ40, p‐tau217, p‐tau181, brain‐derived tau [BD‐tau], glial fibrillary acidic protein [GFAP], and NfL) and growth factors (BDNF and IGF‐1); (ii) the acute effects of a bout of aerobic exercise, resistance exercise, and a resting condition on cognitive and mood outcomes; (iii) whether exercise‐induced changes in CBF mediate changes in blood biomarkers (or vice versa); and (iv) whether exercise‐induced changes in CBF or blood biomarkers mediate changes in cognitive and mood outcomes.

Our *overall hypothesis* is that aerobic exercise will lead to a greater increase in regional CBF compared to resistance exercise (Maass et al. 2015; Pereira et al. 2007), particularly when contrasted with resting, and may also differentially impact blood biomarkers (Vints et al. 2024; Arazi et al. 2021; Alfini et al. 2019).

## 2 Methods

### 2.1 Trial Design and Ethics

**FIGURE 1:**
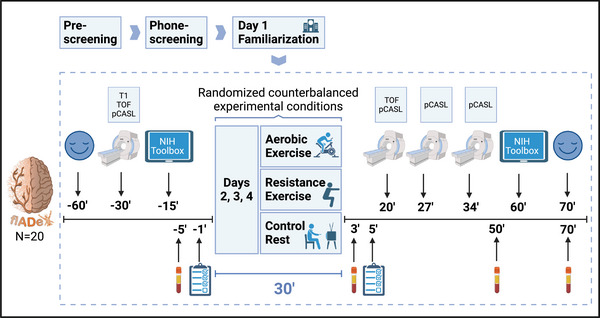
Trial design and key measurement timepoints of the flADex trial. MRI: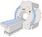; episodic memory and inhibition/attention: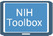; AD and neurodegenerative blood biomarkers and growth factors: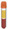; mood (POMS):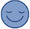; enjoyment (PACES) and feelings of (dis)pleasure (Feeling Scale): 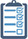 pCASL: Pseudo Continuous Arterial Spin Labeling; T1: T1‐weighted MPRAGE structural; TOF: Time of flight angiography.

The flADex trial will follow a within‐subject crossover design with pretest and posttest assessments in 20 individuals (balanced sex distribution) aged 68–83 years old. Each participant will be included in three experimental conditions (i.e., aerobic exercise, resistance exercise, and resting) in a randomized order (*n* = 3 × 20 observations) (see Figure 1). The trial will be performed in two research institutes at the University of Granada (Spain): (i) the Sports and Health University Research Institute (iMUDS) for the familiarization visit and (ii) the Mind, Brain, and Behaviour Research Centre (CIMCYC) for the experimental condition visits.

The trial, following the principles of the Declaration of Helsinki, has been approved by the Research Ethics Board of the Andalusian Health Service (CEIM/CEI Provincial de Granada; #SICEIA‐2024‐000602; approval: 30/04/2024) and has been registered in ClinicalTrials.gov (NCT06584656; approval: 04/09/2024). All participants will provide written informed consent. Procedure manuals of the flADex trial will be made available to allow other researchers to replicate the results (https://github.com/fladexprojectugr). flADex has been designed following the Standard Protocol Items for Randomized Interventional Trials (SPIRIT) (Chan et al. 2013), and the SPIRIT‐Outcomes 2022 Extension (Butcher et al. 2022) (Table SA1) and is reported in line with the Consolidated Standards of Reporting Trials (CONSORT) 2020 statement for extension to randomized crossover trials (Dwan et al. 2013). Any substantial modifications to the protocol will be communicated to the trial registry, detailed in the main results paper, and submitted for review to the Research Ethics Board.

### 2.2 Eligibility Criteria, Screening of Participants, and Recruitment

**FIGURE 2:**
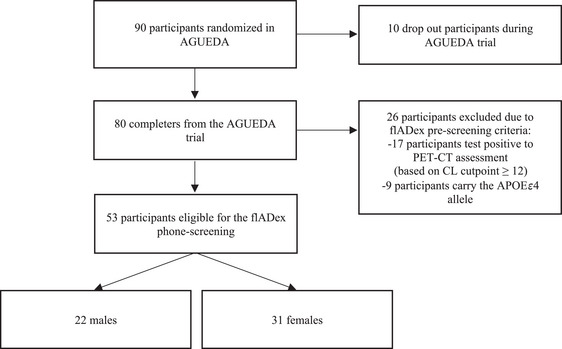
Recruitment and screening of participants for the flADex trial. APOEε4: Apolipoprotein E 4 allele; CL, centiloid; PET‐CT, positron emission tomography—computed tomography.

The flADex trial will recruit individuals from the previous AGUEDA (active gains in brain using exercise during aging) trial (Fernandez‐Gamez et al. 2023; Solis‐Urra et al. 2023). The baseline assessment for the AGUEDA study was conducted between March 2021 and May 2022. The post‐intervention assessment and the final positron emission tomography/computed tomography (PET‐CT) scan were carried out between October 2021 and December 2022. PET‐CT images were acquired using a Biograph Vision 600 Edge digital PET‐CT scanner (Siemens, Erlangen, Germany). The tracer used was [18F]Florbetaben (Neuraceq; Piramal Pharma). Recruitment and screening are illustrated in Figure 2. Eligibility criteria assessed through prescreening and phone screening are detailed in Table 1. Of the 53 eligible individuals from AGUEDA, 20 will be eventually included and randomized for the crossover trial (balanced sex distribution).

**TABLE 1:** Eligibility criteria of the flADex trial.

Inclusion criteria^a^	Exclusion criteria^b^
Adults aged between 68 and 83 years old who participated in the AGUEDA trial.^c^	Ambulatory with pain or regular use of an assisted walking device.
Non‐pathological cerebral beta‐amyloid status (based on a Centiloid cut‐point < 12, measured by PET‐CT).	Not living in community settings during the trial.
APOEε4 negative status.	Pathological diagnosis related to any physical or mental condition that would preclude the participant from exercising or performing the tests.
	MRI incompatibility.

^a^ Prescreening criteria.

^b^ Phone‐screening criteria.

^c^ Participants from the AGUEDA trial were 65 to 80 years old at enrollment and are now 3 years older, making their age range 68–83 years for flADex.

During the prescreening phase, inclusion criteria will be checked using the AGUEDA database. Eligible participants will undergo a phone screening. If no exclusion criteria are identified, they will be scheduled for a familiarization visit (Day 1). This visit will include signing the informed consent form; administering the Montreal Cognitive Assessment (MoCA) for descriptive purposes (Nasreddine et al. 2005); and completing questionnaires related to MRI compatibility, medical history, baseline blood conditions (including fasting state and previous dinner standardization), and physical activity. Weight and height (to calculate body mass index), as well as resting heart rate (HR), will also be measured. Additionally, the exercise protocols will be explained and practiced, and the resistance level of the bands to be used will be recorded. Participants who successfully complete the familiarization visit will be enrolled and randomized (Days 2, 3, and 4), with recruitment set to begin in September 2024.

### 2.3 Randomization

Each participant will be randomized after familiarization with the three trial conditions to achieve a counterbalanced crossover design. A 3 × 3 Latin square design has been implemented for randomization (Keedwell and Dénes 2015), utilizing the “rlatin” function from the “magic” package in R Studio. A randomization dashboard has been created (https://javiersanchez3.shinyapps.io/latin_square_design/) and will be executed for each wave of participants by a blinded researcher external to the trial (Dr. Cabanas‐Sanchez, Autonomous University of Madrid, Spain). To prevent selection bias, allocation concealment will keep the assignment sequence hidden from those enrolling participants, ensuring random and unbiased order assignments.

### 2.4 Blinding

Participants will inherently become aware of their assigned condition at the start of each visit. However, the principal investigator, MRI technician, and research staff responsible for processing MRI data and conducting statistical analyses will remain blinded to minimize researcher bias or expectations.

### 2.5 Overview of Experimental Conditions

**FIGURE 3:**
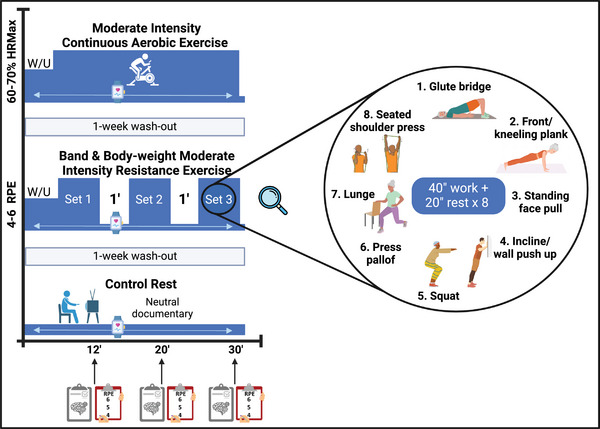
Experimental conditions of the flADex trial. HRMax: Age‐predicted maximum heart rate; RPE: Rating of perceived exertion; W/U: warm‐up.

Each participant's trial will last 3 weeks, preceded by the familiarization visit. Participants will complete three 30‐min experimental conditions, each separated by a 1‐week washout period, with a ratio of one trainer per participant. The aerobic and resistance exercise bouts will include a 4‐min warm‐up and a 26‐min exercise session (see Figure 3). Participants will be instructed to refrain from engaging in any vigorous physical activity within the 48 h preceding the assessments. During the 24 h prior to each assessment, they must also avoid moderate‐to‐vigorous physical activity, the consumption of stimulants such as caffeine, and alcohol intake. All conditions will be performed following a 12‐h overnight fast after the consumption of a standardized dinner the previous evening.

#### 2.5.1 Moderate Aerobic Exercise Condition

Participants will perform continuous moderate‐intensity aerobic exercise on a cycle ergometer (Keyser M3) at 60%–70% of their age‐predicted maximal HR (HRmax; calculated as 220 ‐ age) (Zelazo et al. 2013). To achieve the target HR, the intensity will be adjusted according to the participant's response, with a primary focus on increasing the ergometer's resistance rather than the cadence. Beta‐blocker use will be documented through the administration of two questionnaires: a medical information questionnaire administered during the familiarization session and a condition‐day checklist used to record any medications taken on the day before each assessment. Sensitivity analyses will be conducted, if necessary, to account for the potential impact of beta blockers on HR‐based exercise prescription. For further details, refer to Supporting Information A.2.1.

#### 2.5.2 Resistance Exercise Condition

Participants will perform a combination of upper‐ and lower‐body exercises using elastic bands with different resistance levels and their body weight as primary resistance. The target intensity will be based on 4–6 points of the Rate of Perceived Exertion (RPE, 0‐10) using the OMNI‐Resistance Exercise Scale (Morishita et al. 2019), although HR will also be recorded. Participants will perform three sets of eight different exercises, with each exercise lasting 40 s, followed by 20 s of rest and a 1‐min rest period between sets. Exercises can be adapted according to each participant's ability or situation (e.g., bloodline placement). The exercises will include glute bridge, front plank, standing face pull, incline push‐up, squat, press pallof, walking lunge, and seated shoulder press. See Supporting Information A.2.2 for extended information.

#### 2.5.3 Resting Condition

Participants will remain seated and isolated in a room, watching a standardized neutral documentary on a tablet for 30 min without any cognitive engagement (https://youtu.be/WLeTofB2wbo?feature=shared).

### 2.6 Outcomes

Table 2 summarizes the primary and secondary outcomes and exercise‐related variables, along with the assessment instruments and data collection time points.

**TABLE 2:** Outcomes, instruments, and time points of data collection of the flADex trial.

**Cerebral blood flow**	−30ʹ	—	20ʹ, 27ʹ, and 34ʹ	MRI: turbo gradient spin echo‐pseudo continuous arterial spin labeling (pCASL) mL/100 g/min
**Mood status**	−60ʹ	—	70ʹ	Scale Profile of Mood States (POMS) 15‐item scale Total mood disturbance: 90–144 total score
**Episodic memory**	−15ʹ	—	60ʹ	NIH Toolbox: Picture Sequence Memory Test Raw score n total correct items
**Inhibition/attention**	−15ʹ	—	60ʹ	NIH Toolbox: Flanker test Incongruent trials inverse efficiency score
**Blood biomarkers of AD pathology and neurodegeneration**	−5ʹ	—	3ʹ, 50ʹ, and 70ʹ	Plasma samples Aβ42/40 ratio, p‐tau217, p‐tau181, BD‐tau, GFAP, and NfL pg/mL
**Growth factors**	−5ʹ	—	3ʹ, 50ʹ, and 70ʹ	Serum samples BDNF, IGF‐1 pg/mL (BDNF), ng/mL (IGF‐1)
**Emotional response**	−1ʹ	—	1ʹ	Feeling scale 11‐points scale −5 to 5 total score
**Rate of perceived exertion (only aerobic and resistance condition)**	—	12ʹ, 20ʹ, and 30ʹ	—	OMNI‐Resistance Exercise Scale (OMNI‐RES) 10‐point scale 1–10 total score
**Cognitive engagement**	—	12ʹ, 20ʹ, and 30ʹ	—	Cognitive Load Measurement Scale 9‐point scale 1–9 total score
**Heart rate**	—	During 30ʹ	—	Polar H10 Heart rate per minute beats/min
**Repetitions in reserve (only resistance condition)**	—	After each exercise	—	The Repetitions in Reserve (RIR) Number of repetitions n
**Enjoyment**	—		5ʹ	Physical Activity Enjoyment Scale (PACES) 8‐items scale 7–56 total score
**Blood pressure**	−60ʹ		1ʹ	Omron M3 Systolic and diastolic blood pressure millimeters of mercury (mmHg)
**Temperature**	−50ʹ		20ʹ	Digital Infrared Forehead Thermometer (iPiccoli) Temperature degrees Celsius (°C)

Abbreviations: Aβ42/40, ratio Amyloid‐beta 42/Amyloid‐beta 40; BDNF, Brain‐Derived Neurotrophic Factor; BD‐tau, Brain‐Derived tau; GFAP, Glial Fibrillary Acidic Protein; IGF‐1, Insulin‐Like Growth Factor 1; MRI, Magnetic Resonance Imaging; NfL, Neurofilament Light Chain; p‐tau217, p‐tau181, phosphorylated tau protein at positions 217 and 181.

#### 2.6.1 Primary Outcomes

*Change in CBF*: CBF will be assessed by MRI using a Siemens Magnetom PRISMA Fit 3T scanner with a 64‐channel head coil located at CIMCYC. Specific acquisition parameters for MRI are detailed in Table 3. Body temperature and any incidental bodily conditions will be registered before MRI acquisitions to ensure physiological stability, as factors such as fever may affect CBF measurements. Pseudo‐continuous Arterial Spin Labeling (pCASL) sequences are used to determine global and regional CBF in resting supine position conditions. A structural T1 sequence (only pre‐condition) will be used to co‐register the pCASL and delineate regions of interest for CBF. Time‐of‐flight angiography (TOF) (before pCASL pre‐condition and before first pCASL post‐condition) sequence will be used to identify the carotid arteries. pCASL measurements will be taken 30 min prior to the start of the condition and at 20, 27, and 34 min following the completion of the condition. These time points represent the start of each pCASL sequence, each lasting approximately 7 min. The timing of the postexercise CBF assessments was selected to capture the temporal dynamics of CBF following the experimental condition. Our aim is to obtain a detailed picture of CBF variation during the immediate recovery period. The selected time points were determined by considering the necessary transition and preparation intervals between the end of the condition and the start of each CBF measurement. Importantly, we prioritized initiating the first post‐condition measurement as early as possible while allowing sufficient time to ensure participant safety, repositioning, and imaging setup (Palmer et al. 2023). The preprocessing analysis of pCASL will be carried out using the most up‐to‐date pipelines (ASLprep).

**TABLE 3:** MRI parameters for the flADex trial.

Sequence	Parameters	Measure	Acquisition time (min)
TGSE pCASL	Transversal resolution = 2.5 mm × 2.5 mm × 3.0 mm, TR = 4100 ms, TE = 36.8 ms, Background suppression, M0 and 12 label‐control pairs, Postlabeling delays = [500, 500, 1000, 1000, 1500, 2000, 2000, 2000, 2500, 2500, 2500 ms], labeling duration = 1500 ms, 4 segment readout	Cerebral blood flow	7
T1‐weighted MPRAGE structural	Sagittal, 0.8 mm isotropic resolution, TR = 2400 ms, TE = 2.31 ms, TI = 1060 ms, FOV = 256 mm, slices = 224	Brain structure	6
TOF (Time of flight angiography)	Resolution = 0.8 × 0.8 × 1.2 mm^3^, TR = 19 ms, TE = 2.82 ms, flip angle = 18 grad, FOV = 200 mm, acquisition bandwidth = 219 Hz/Px, slices = 32 (oversampling 25%)	Angiography (cerebral vascularization)	1

Abbreviations: FOV, field of view; MPRAGE, Magnetization prepared rapid gradient echo; TE, echo time; TGSE pCASL, turbo gradient spin echo pseudo‐continuous arterial spin labeling; TI, inversion time; TR, repetition time.

#### 2.6.2 Secondary Outcomes

*Changes in blood biomarkers of AD pathology and neurodegeneration and growth factors*: Aβ42, Aβ40, p‐tau217, p‐tau181, BD‐tau, GFAP, NfL, BDNF, and IGF‐1 will be assayed. Blood samples will be collected at the CIMCYC facilities by a qualified nurse. Each day will involve four draws with one puncture; one draw 5 min pre‐condition (using a bloodline), followed by one draw 3 min after the condition, and draws at 50 and 70 min after the condition (Huber et al. 2023). The timing of the postexercise blood draws was designed to capture the acute temporal profile of blood biomarkers related to AD pathology, neurodegeneration, and neurotrophic growth factors. Our rationale was to obtain an immediate postexercise sample to reflect the initial physiological response, followed by additional samples during the recovery period to monitor changes over time. The selected time points were informed by prior literature indicating different release kinetics for these biomarkers (Huber et al. 2023), as well as by practical considerations related to the time required for participants to complete the condition and be properly prepared for blood collection. Additionally, the timing of the second and third postexercise blood draws (i.e., T2 and T3) was constrained by the MRI schedule, as these blood samples were collected immediately following the corresponding post‐condition CBF assessments. Based on previous acute studies, we expect the peak change from pre‐ to post‐condition of some neurotrophic and growth factors to happen immediately after exercise (within 5 min of exercise) (Huber et al. 2023); thus, a 3‐min time point was chosen. Since there is some variability in previous literature, and the precise kinetics may vary depending on exercise intensity, we also included 50‐ and 70‐min time points.

During each session, a total of 48 mL of blood will be collected (4 mL per tube × 3 tubes per draw × 4 draws). Each draw will include two ethylenediaminetetraacetic acid (EDTA) tubes for plasma and one serum separator tube (SST) with clot activator and gel. Samples will be processed according to regulations (Zeng et al. 2024). Following collection, blood samples will be centrifuged at 3000 rpm at room temperature for 10 min (serum tubes need 40 min before centrifugation). The resulting plasma and serum will be aliquoted into 0.65 mL Eppendorf tubes, pseudo‐anonymized, and stored at −80°C at iMUDS for analysis upon trial completion. All blood collection and processing will be conducted at the same location. Blood biomarkers of AD pathology and neurodegeneration will be measured using single molecular array (Simoa) following the manufacturer's instructions at the University of Pittsburgh (Ashton et al. 2022).

*Change in episodic memory*: The Picture Sequence Memory Test from the Cognitive NIH Toolbox (a computer‐based battery that has been validated in Spanish) measures episodic memory. The raw score from the cumulative number of adjacent pairs of pictures remembered correctly over two learning trials will be used as the outcome (Zelazo et al. 2013).

*Change in inhibition/attention*: The Flanker test from the Cognitive NIH Toolbox measures inhibitory control and attention. The outcome will be the inverse efficiency score of incongruent trials calculated as reaction time/accuracy (RT/ACC) (Zelazo et al. 2013).

*Mood status*: A shortened version of the Profile of Mood States (POMS) scale will be used to assess mood status (Fuentes et al. 1994). It consists of adjectives or mood descriptors, where individuals rate how they have been feeling on a scale typically ranging from *Not at all* (0) to *Extremely* (4). The shortened version includes 15 items divided into five dimensions: depression, vigor, anger, tension, and fatigue. The final score, calculated as [depression] + [anger] + [tension] + [fatigue] − [vigor], reflects total mood disturbance, with higher scores indicating more negative emotions and less positive energy.

*Emotional response*: The feeling scale (Vázquez‐Morejón Jiménez et al. 2004) will be used to assess emotional response. It is an 11‐point scale ranging from −5 (*very bad*) to +5 (*very good*) used to measure an individual's feelings in terms of pleasure or displeasure.

#### 2.6.3 Exercise‐Related Variables

*Rate of Perceived Exertion*: Rate of Perceived Exertion (RPE) will be assessed using the OMNI‐Resistance Exercise Scale (OMNI‐RES) of perceived exertion from 0 to 10 points (Morishita et al. 2019).

*Cognitive Engagement*: The cognitive load scale is a 9‐item scale used to assess the mental effort or cognitive load experienced by individuals when engaging in a task, particularly in educational or learning contexts. It involves a self‐reported measure where participants rate their perceived mental effort on a scale ranging from 1—*very low mental effort* to 9—*very high mental effort* (Ouwehand et al. 2021; Paas et al. 2003).

*Heart Rate*: HR will be continuously monitored second‐by‐second during all three conditions using a Polar H10 monitor, which includes a chest strap and wristwatch.

*Repetitions in Reserve (RIR)*: RIR is a self‐regulation technique used in resistance training to gauge exercise intensity. It involves estimating the number of additional repetitions a participant could perform before reaching muscle failure after completing a set (Bastos et al. 2024).

*Enjoyment*: Enjoyment of physical activity will be assessed using the reduced and validated version of the physical activity enjoyment scale (PACES) (Mullen et al. 2011), an 8‐item scale assessing in a range of 1–7 a series of sensations or moods with respect to their opposites.

*Blood Pressure (BP)*: BP will be measured using a validated automated monitor (Omron M3, Intellisense, OMRON Healthcare Europe, Spain) with participants seated and their left arm at heart level. After 5 min of rest, two readings will be taken at 1‐min intervals, and the average of the readings will be used for analysis.

*Temperature*: Body temperature will be measured using a digital infrared forehead thermometer (iPiccoli, AET‐R1B1), with participants standing, ensuring their forehead is clean and free from sweat.

### 2.7 Safety and Adverse Events

The expected risks and side effects from participating in the flADex trial are low (Alfini et al. 2019; Kleinloog et al. 2019; Vints et al. 2024). Any unusual symptoms (e.g., dizziness, chest pain) or adverse events during the trial will be noted by the research staff. Participants may withdraw from the trial at any time, and the research staff may terminate a participant's involvement if any minimal risk to their safety is identified. Extended information can be found in Supporting Information A3.

### 2.8 Sample Size

Previous studies on acute exercise have shown that a single bout of aerobic exercise changed CBF by 15% (Mast et al. 2022) and 9% (Macintosh et al. 2014). Specifically, the sample size is based on the mean changes (M) in CBF of 6 mL/100 g/min (*M*1 = 40.5 and *M*2 = 34.2) with a standard deviation (SD) of 6.46 mL/100 g/min (Long and Holtzman 2019). Considering the calculation of Cohen's *d* based on previous studies (*d* = *M*1−*M*2/combined SD), a large effect size (Cohen's *d* = 0.9) is expected (Long and Holtzman 2019). Therefore, using an alpha of 0.05 and a standard power of 80%, a sample size of 20 is needed. Participants who withdraw from the trial will be replaced to ensure sufficient power to assess our primary outcome. Thus, the experiment will be completed when 20 participants have completed all three experimental conditions. If a participant misses one visit, it will be rescheduled within 2 weeks of the last completed visit. This sample size is feasible based on our previous experiences involving MRI and exercise interventions.

### 2.9 Data Analysis Plan

#### 2.9.1 Brief Description of the Primary and Secondary Analyses

*Effects on Primary and Secondary Outcomes*: The primary and secondary outcomes will be analyzed following a per‐protocol approach using a linear mixed model. The model will include fixed effects for time (four or two levels depending on the outcome), condition (three levels), and time*condition interaction, and the unique participant identifier as a random effect. The number and patterns of missing data will be explored and reported. Missing data will be assumed to be missing at random and handled within the linear mixed model analyses. Model assumptions will be assessed. If violations are detected, appropriate corrective measures will be implemented, including data transformations. Condition effects will be evaluated using a time‐by‐treatment interaction term and described with estimated marginal means and 95% confidence intervals. All statistical tests will be two‐tailed. *p* for significance will be set at 0.05.

*Mediation and correlation analysis*: Mediation analysis will be performed following AGReMA (A Guideline for Reporting Mediation Analyses) recommendations (Milà‐Alomà et al. 2022), using CBF and blood biomarkers as mediators depending on the outcome. When blood biomarkers are the outcomes, CBF will act as the mediator, and vice versa. When cognitive and mood indicators are the outcomes, both CBF and blood biomarkers will act as mediators. In addition, bivariate correlations will be performed between changes in CBF, changes in blood biomarkers, and changes in cognitive and mood outcomes.

*Exercise intervention parameters*: Parameters related to the exercise conditions (i.e., RPE, cognitive engagement, HR, RIR, and enjoyment) will be presented in a descriptive manner. These variables will not be used as covariates in the main models but will help interpret differences across types of exercise.

While the study compares the effects of different conditions (aerobic vs. resistance vs. resting) on CBF, blood biomarkers, and cognitive and mood outcomes, we acknowledge the intrinsic differences between exercise types, such as intensity, energy expenditure, and cognitive engagement. Exercise‐related variables will be descriptively compared across conditions. When relevant, they may be included as covariates in secondary or exploratory models to explore whether they account for variability in outcome measures. However, our primary intention is not to control for or eliminate these natural differences but rather to characterize how these distinct types of exercise affect the outcomes, with all their inherent features. This approach supports the validity of the intervention and aligns with the aim of exploring the real effects of different exercise strategies.

#### 2.9.2 Exploratory Analyses

An exploratory moderation analysis will assess whether sex moderates the acute effects of different exercise types (aerobic vs. resistance vs. resting) on CBF. These results will be interpreted with caution due to sample size limitations but are expected to offer insights into individual variability in physiological responses to exercise.

### 2.10 Data Management and Sharing

A comprehensive data management plan has been developed, ensuring proper handling, storage, and sharing of data in accordance with ethical and legal guidelines. Extended information can be found in Supplementary material A4.

## 3 Discussion

The flADex trial seeks to advance the understanding of the acute effects of different exercise types on CBF, blood biomarkers, and cognitive function in older adults. The findings may reveal mechanisms through which exercise delays or slows cognitive decline, particularly vascular and molecular pathways associated with AD pathology in aging populations at risk for the disease.

flADex is expected to reveal distinct CBF response patterns between aerobic and resistance exercise. A systematic review (*n* = 52 studies) showed that during a bout of aerobic exercise, CBF generally follows an inverted U‐shaped response (Mulser and Moreau 2023). This autoregulatory mechanism ensures the increased oxygen and nutrient demands during exercise are met while preventing vascular overloading, which is particularly relevant in aging populations prone to cerebrovascular dysregulation (Palmer et al. 2023; Perdomo et al. 2019). Additionally, acute increases in CBF, particularly in the hippocampus—a critical region for memory—have been observed following aerobic exercise (Palmer et al. 2023) and 15, 40, and 60 min after exercise cessation (Steventon et al. 2020), suggesting enhanced vascular plasticity and a potential protective effect against cognitive decline. However, evidence on acute CBF changes following other types of exercise (e.g., resistance training) remains limited—particularly across different brain regions, where fluctuations are expected to vary regionally (Palmer et al. 2023), highlighting the need for further investigation.

Chronic aerobic and resistance exercise have complementary effects on brain health. Aerobic exercise supports gray matter preservation, reduces systemic inflammation, and improves lipid profiles (Pascual‐Lucas et al. 2023; Milà‐Alomà et al. 2022), while resistance training benefits white matter integrity and influences muscle‐related biomarkers like creatine kinase and anabolic hormones (e.g., testosterone and IGF‐1) (Kleinloog et al. 2019; Voss et al. 2016). Acutely, both exercise types trigger a transient increase in pro‐inflammatory cytokines and oxidative stress markers. However, this response is part of a hormetic process that, in turn, stimulates the production of anti‐inflammatory cytokines and endogenous antioxidants. Aerobic exercise has been shown to more effectively enhance insulin sensitivity and lipid metabolism, while resistance training induces a more pronounced inflammatory response due to muscle tissue remodeling (Maass et al. 2015; Fernandez‐Gamez et al. 2023; Perdomo et al. 2019). flADex will provide novel insights into the distinct and overlapping mechanisms through which exercise acutely impacts CBF and its cognitive implications in older adults. Understanding these acute effects could help optimize chronic exercise interventions tailored to individual cerebrovascular and metabolic needs.

Beyond CBF, flADex will measure changes in blood biomarkers of AD pathology and neurodegeneration and growth factors, which are critical to understanding AD and brain health. For instance, emerging evidence suggests that aerobic exercise may acutely reduce the levels of NfL, a marker of axonal damage and neuronal injury (Joisten et al. 2021), and modulate the kynurenine pathway, enhancing the production of neuroprotective metabolites (Schultz et al. 2020). Similarly, resistance exercise, despite inducing greater oxidative stress and inflammation due to muscle repair processes, might also promote adaptive mechanisms that contribute to neuroprotection (Joisten et al. 2021). BDNF and IGF‐1 are also vital for brain health, as they support neuronal survival and synaptic growth and aid in brain repair and cognitive function, respectively. Acute aerobic and resistance exercise significantly increase serum BDNF and IGF‐1 concentrations postexercise in older men (60 mean age) compared to controls (Arazi et al. 2021). Measuring these biomarkers will help elucidate how acute exercise‐induced changes impact neuronal integrity, inflammation, and cognition, thereby advancing our understanding of the molecular pathways through which exercise may support AD prevention and management during aging. Additionally, examining the acute effects of exercise on biomarkers of AD and neurodegeneration will help determine whether blood biomarker sampling should be performed under standardized resting conditions prior to measurements to improve diagnostic accuracy.

Participants with positive brain amyloid status were excluded from the flADex trial to minimize the influence of underlying AD pathology on cerebrovascular and biomarker responses (Rodriguez‐Ayllon et al. 2024; Ashton et al. 2022; Milà‐Alomà et al. 2022). In addition, individuals carrying the APOE4 allele were excluded due to their known genotype‐specific alterations in CBF regulation (Zlokovic 2011; Korte et al. 2020) and inflammatory and neurotrophic responses (Long and Holtzman 2019; Ashton et al. 2022). Including these participants could have introduced significant heterogeneity, as both amyloid positivity and APOE4 status are associated with early neurodegenerative changes and may modify the acute response to exercise. Their presence could mask the specific effects of different exercise types in a sample of older adults (Alfini et al. 2019; Pascual‐Lucas et al. 2023). By excluding these two high‐risk profiles, the study aimed to reduce biological variability and increase the specificity of the findings (Rodriguez‐Ayllon et al. 2024; Milà‐Alomà et al. 2022). Future trials should explore whether APOE4 carriers, compared to noncarriers, respond differently to specific exercise interventions (Zlokovic 2011; Long and Holtzman 2019).

It is also important to note that the flADex trial will include a balanced sex distribution, which allows us to explore potential sex‐related differences in the acute responses to exercise. Prior research has shown that females generally exhibit higher resting CBF than males (Zlokovic 2011). Including both sexes equally ensures that findings are more generalizable and may help identify whether certain exercise types have sex‐specific effects on CBF and related outcomes (Maass et al. 2015; Palmer et al. 2023). Future studies with larger sample sizes should consider whether sex moderates acute physiological responses to different exercise types.

The flADex trial has limitations, including limited sample size and potential confounding factors such as diet. The exercise types were not matched for caloric expenditure or intensity, which may affect acute systemic responses—for instance, neurotrophic factors may be influenced by energy demand and glycogen depletion (Arazi et al. 2021). Lastly, although we attempted to minimize practice effects by using different versions of the Picture Sequence Memory Test across sessions, the short interval between assessments may still have introduced learning effects that could influence cognitive outcomes. The flADex trial also has several strengths. Its crossover design enhances efficiency and reduces variability by using participants as their own controls. Advanced imaging and biomarker analyses provide detailed insights into exercise effects on CBF and neurovascular responses. Comparing aerobic and resistance exercises may also highlight distinct acute responses. Lastly, this study is crucial for recommending standardized rest conditions prior to blood sample collection when using biomarkers for diagnosing, monitoring disease progression, and evaluating drug responses (Huber et al. 2023).

In conclusion, the flADex trial will address critical research gaps by investigating the acute effects of resistance exercise on CBF, blood biomarkers, and cognitive function, areas where evidence remains limited compared to aerobic exercise. This study will provide novel insights into how different exercise types acutely influence brain health and neuroprotection during aging.

## Author Contributions

**Isabel Martín‐Fuentes**: conceptualization, investigation, writing – original draft, methodology, visualization, writing – review and editing, project administration. **Beatriz Fernandez‐Gamez**: investigation, writing – review and editing, methodology. **Sol Vidal‐Almela**: writing – review and editing, methodology. **Alfredo Caro‐Rus**: investigation, writing – review and editing. **Patricio Solis‐Urra**: writing – review and editing, methodology, conceptualization. **Lucía Sánchez‐Aranda**: writing – review and editing, methodology, investigation. **Javier Fernández‐Ortega**: writing – review and editing, methodology, investigation. **Javier Sanchez‐Martinez**: methodology, writing – review and editing, investigation. **Andrea Coca‐Pulido**: writing – review and editing, methodology, investigation. **Marcos Olvera‐Rojas**: writing – review and editing, methodology, investigation. **Emilio J. Barranco‐Moreno**: writing – review and editing, investigation. **Jose D. Marin‐Alvarez**: writing – review and editing, investigation. **Esmée A. Bakker**: writing – review and editing, conceptualization, methodology. **Angel Toval**: writing – review and editing, conceptualization. **Darío Bellón**: writing – review and editing. **Alessandro Sclafani**: writing – review and editing. **Thomas K. Karikari**: conceptualization, methodology, writing – review and editing. **Kirk I. Erickson**: conceptualization, writing – review and editing, methodology. **Manuel Gómez‐Río**: writing – review and editing, methodology, conceptualization. **Francisco B. Ortega**: methodology, writing – review and editing, conceptualization. **Irene Esteban‐Cornejo**: conceptualization, investigation, funding acquisition, writing – review and editing, visualization, methodology, project administration, resources, supervision.

## Ethics Statement

The trial, which has been registered in ClinicalTrials.gov (NCT06584656; approval: 04/09/2024), will follow the principles of the Declaration of Helsinki and has been approved by the Research Ethics Board of the Andalusian Health Service (CEIM/CEI Provincial de Granada; #SICEIA‐2024‐000602; approval: 30/04/2024).

## Consent

All participants will provide written informed consent approved by the Research Ethics Board of the Andalusian Health Service.

## Conflicts of Interest

TKK has consulted for Quanterix Corporation, SpearBio Inc., Neurogen Biomarking LLC., and Alzheon, has served on advisory boards for Siemens Healthineers, Neurogen Biomarking LLC. and Alzheon (which may come with minority stock equity interest/stock options), outside the submitted work. He has received in‐kind research support from Janssen Research Laboratories, SpearBio Inc., and Alamar Biosciences, as well as meeting travel support from the Alzheimer's Association and Neurogen Biomarking LLC., outside the submitted work. TKK has received royalties from Bioventix for the transfer of specific tau antibodies and assays to third party organizations. He has received honoraria for speaker/grant review engagements from the NIH, UPENN, UW‐Madison, the Cherry Blossom symposium, the HABS‐HD/ADNI4 Health Enhancement Scientific Program, Advent Health Translational Research Institute, Brain Health conference, Barcelona‐Pittsburgh conference, the International Neuropsychological Society, the Icahn School of Medicine at Mount Sinai and the Quebec Center for Drug Discovery, Canada, all outside of the submitted work. TKK serves/has served as a guest editor for npj Dementia, as an invited member of the World Health Organization committee to develop preferred product characteristics for blood‐based biomarker diagnostics for Alzheimer's disease, as an executive committee member for the Human Amyloid Imaging (HAI) conference, as an elected member of the NACC ADRCs Steering Committee, as co‐director of the NACC ADRCs Biofluid Biomarker Working Group, and as a member of the Alzheimer's Association committees to develop Appropriate Use Criteria for clinical use of blood‐based biomarkers, and treatment related amyloid clearance. TKK is an inventor on several patents and provisional patents regarding biofluid biomarker methods, targets and reagents/compositions, that may generate income for the institution and/or self should they be licensed and/or transferred to another organization. These include WO2020193500A1: Use of a ps396 assay to diagnose tauopathies; 63/679,361: Methods to Evaluate Early‐Stage Pre‐Tangle TAU Aggregates and Treatment of Alzheimer's Disease Patients; 63/672,952: Method for the Quantification of Plasma Amyloid‐Beta Biomarkers in Alzheimer's Disease; 63/693,956: Anti‐tau Protein Antigen Binding Reagents; and 2450702‐2: Detection of oligomeric tau and soluble tau aggregates.

## Peer Review

The peer review history for this article is available at https://publons.com/publon/10.1002/brb3.70636

## Supporting information




**Supplementary Material**: brb370636‐sup‐0001‐SuppMat.docx

## Data Availability

Extended information about the protocols is available in the GitHub repository https://github.com/fladexprojectugr.
